# Nasal Resistome Development in Infants With Cystic Fibrosis in the First Year of Life

**DOI:** 10.3389/fmicb.2019.00212

**Published:** 2019-02-26

**Authors:** Aurélie Allemann, Julia G. Kraemer, Insa Korten, Kathryn Ramsey, Carmen Casaulta, on Behalf of SCILD Study Group, Daniel Wüthrich, Alban Ramette, Andrea Endimiani, Philipp Latzin, Markus Hilty

**Affiliations:** ^1^Institute for Infectious Diseases, University of Bern, Bern, Switzerland; ^2^Graduate School for Cellular and Biomedical Sciences, University of Bern, Bern, Switzerland; ^3^Institute for Work and Health (IST), University of Lausanne and University of Geneva, Epalinges, Switzerland; ^4^Division of Respiratory Medicine, Department of Paediatrics, Inselspital, University of Bern, Bern, Switzerland; ^5^Applied Microbiology Research Unit, Department of Biomedicine, University of Basel, Basel, Switzerland; ^6^Division of Clinical Microbiology, University Hospital Basel, Basel, Switzerland

**Keywords:** functional metagenomics, nasal swabs, beta-lactamases, resistance, antibiotics

## Abstract

Polymicrobial infections of the respiratory tract due to antibiotic resistant bacteria are a great concern in patients with cystic fibrosis (CF). We therefore aimed at establishing a functional metagenomic method to analyze the nasal resistome in infants with CF within the first year of life. We included samples from patients before antibiotic treatment, which allowed obtaining information regarding natural status of the resistome. In total, we analyzed 130 nasal swabs from 26 infants with CF and screened for β-lactams (ampicillin, amoxicillin-clavulanic acid, and cefuroxime) and other classes of antibiotic resistances (tetracycline, chloramphenicol and trimethoprim-sulfamethoxazole). For 69 swabs (53% of total), we found at least one non-susceptible phenotype. Analyses of the inserts recovered from non-susceptible clones by nanopore MinION sequencing revealed a large reservoir of resistance genes including mobile elements within the antibiotic naïve samples. Comparing the data of the resistome with the microbiota composition showed that the bacterial phyla and operational taxonomic units (OTUs) of the microbiota rather than the antibiotic treatment were associated with the majority of non-susceptible phenotypes in the resistome. Future studies will reveal if characterization of the resistome can help in clinical decision-making in patients with CF.

## Introduction

The respiratory tract microbiome is a complex ecosystem that evolves from birth to adulthood and may be disordered during disease or antibiotic (AB) therapy (Bomar et al., 2017). Its composition is influenced by many maternal and environmental factors, including birth mode and breast feeding during the first months of life (Bogaert et al., 2011; Biesbroek et al., 2014a; Bosch et al., 2016). Studies using 16S rRNA sequencing demonstrated that accelerated microbiota maturation was associated with microbiota instability, a marker of susceptibility to respiratory tract infections (RTI) (Biesbroek et al., 2014b; Bosch et al., 2017). In a previous study, we prospectively profiled the nasal bacterial microbiota composition in infants with cystic fibrosis (CF) (Mika et al., 2016). Nasal swabs from infants with CF were collected every second week during the first year of life (Mika et al., 2015). As patients with CF suffer from frequent bacterial infections, the impact of AB therapy early in life on the microbiota composition was assessed (Mika et al., 2016; Prevaes et al., 2016). Analyses showed a decrease in *Staphylococcus aureus* at and after antibiotic treatment while coagulase-negative Staphylococci increased.

However, little is known about the antimicrobial susceptibility profiles of the whole respiratory microbiota in CF. As patients with CF are exposed to multiple courses of antibiotics, the emergence and selection of AB resistant strains is an important concern (Sherrard et al., 2014; Cuthbertson et al., 2016). In the case of polymicrobial infections, β-lactamase-producing bacteria may protect β-lactams-susceptible members of the community by releasing these enzymes in the environment and inactivating antimicrobial agents (Brook, 2004; Stiefel et al., 2015). In addition, β-lactamases-positive *Prevotella* spp. has been identified to be highly prevalent in patients with CF and could passively protect the pathogen *Pseudomonas aeruginosa* against the antimicrobial effects of ceftazidime (Sherrard et al., 2016).

Culture-based methods for pathogen identification followed by antimicrobial susceptibility testing are routinely performed in clinical laboratories. However, a considerable amount of commensal or potentially pathogenic bacteria of the microbiota are not routinely screened for AB resistance (Grice and Segre, 2011). Therefore, the antimicrobial susceptibility of the entire bacterial community is often neglected and shotgun metagenomics sequencing technologies have been recently used for the detection of resistance genes within patients with CF (Lim et al., 2014). Results from a very recent study using shotgun metagenomics sequencing indicated that the microbiome of CF patients with low pulmonary function is enriched in genes encoding efflux-mediated antibiotic resistance mechanisms (Bacci et al., 2017). However, it has been acknowledged that the application of metagenomics sequencing to large patient cohorts can be very expensive and that combining metagenomic screening of pooled DNA extracts with validatory quantitative PCR-based analyses may have the potential to provide important insights into the resistome in CF (Taylor et al., 2018). However, shotgun sequencing data relies on databases which, even if well maintained, may not reflect the entity of all resistance genes. In addition, it is unclear if resistance genes derived by shotgun sequencing express the actual resistance phenotype. In contrast, functional metagenomics, which is also a culture-independent method, enables the phenotypic identification of antimicrobial resistance genes (ARG) in bacterial genomes, including those that are currently unknown (Sommer et al., 2009; Mullany, 2014; van der Helm et al., 2017).

In this study, we aimed at setting up and establishing a functional metagenomic approach to describe the nasal resistome in infants with CF during the first year of life. Furthermore, we have aimed at more specifically investigating how the composition of the resistome is associated with antibiotic treatment and bacterial composition of the microbiota in patients with CF.

## Materials and Methods

### Samples, Study Design and Participants

During 2011–2014, nasals swabs were collected from infants with CF enrolled in the prospective Swiss Cystic Fibrosis Infant Lung Development (SCILD) cohort. The SCILD cohort includes infants with CF diagnosed following new-born screening from CF centers throughout Switzerland and was established in 2011 (Kieninger et al., 2017). Parents collected anterior nasal swabs from 26 infants every second week during their first year of the infant’s life (Mika et al., 2016; Korten et al., 2018). We selected all samples with more than 40 ng/μL of PCR product after 16S rRNA PCR and subsequent purification from our previous study (Mika et al., 2016). We therefore analyzed 130 samples from 26 infants using functional metagenomics. Detailed clinical information regarding the patients was collected including age (months) at swab collection and complete AB history (Supplementary Table 1). The study was approved by the Ethics Committee of the Canton of Bern, Switzerland. Written informed consent was obtained from the parents of the participants in this study.

### DNA Preparation and Functional Metagenomic Assays

**FIGURE 1:**
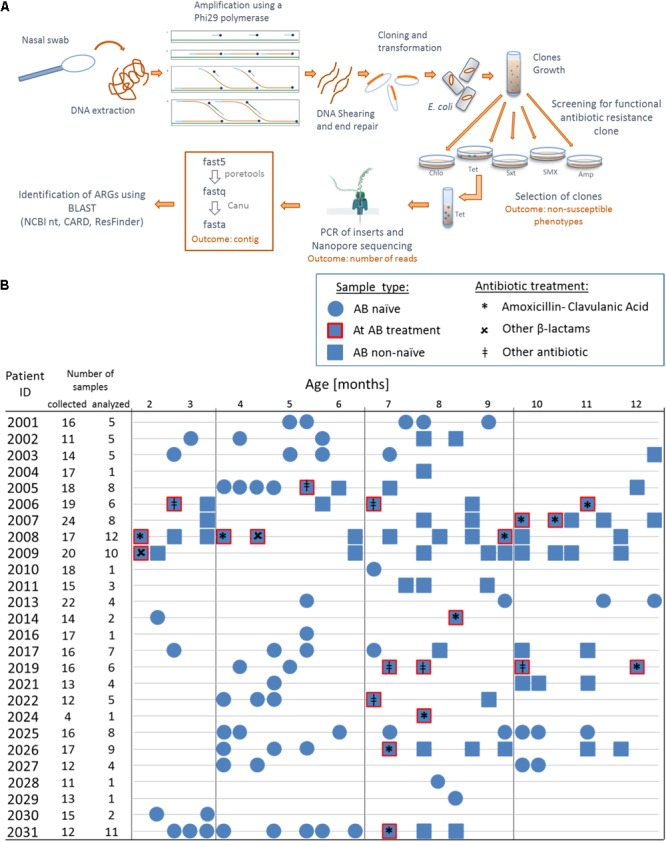
Overview of the functional metagenomic approach, samples collection and patient history with antibiotic therapy. **(A)** Schematic representation of the experimental workflow. **(B)** We used 130 nasal swabs from 26 infants with CF enrolled in a cohort study. We chose samples containing a decent amount bacterial DNA (≥40 ng/μL 16S rRNA PCR product) for functional metagenomic analysis. Samples are represented according to patient antibiotic history at the time of sampling; circles: antibiotic naïve, squares with red border: during an antibiotic treatment and squares: after one or more antibiotic therapy.

DNA was extracted from 200 μL of transport medium of nasal swab using QIAamp DNA Mini Kit (Qiagen) following provider’s instructions. To enrich our samples, whole genomic DNA was amplified using a multiply-primed rolling circle amplification approach (Dean et al., 2001): 10 U of φ29 polymerase (NEB, cat. No. M0269L) were used to amplify 5 μL of extracted DNA with a final concentration of 50 μM random primers (Promega, C1181) and 2mM dNTPs (Figure 1A). After shearing with Megaruptor 2 (Diagenode), fragments between 2 and 9 kb were recovered, purified with AMPure XP beads (Beckmann-Coulter) and end-repaired (NEB, E5060L, following provider’s instructions) in reactions of 50 μL, with incubation for 40 min at room temperature. After heat inactivation for 15 min at 70°C, end-repaired DNA (2.5 μL, 20–100 ng/μL) was ligated into pCR-Blunt (0.5 μL, 25 ng/μL) vector (Thermo Fisher Scientific, K270040, following provider’s instructions, 5 μL reaction volume) for 5 h at 20°C before heat inactivation at 70°C for 20 min. We then transformed 2 × 25 μL NEB 10-beta electrocompetent *E. coli* (DH10B) cells with 2 × 2 μL of the ligation product to create two cells libraries for each sample (two libraries for reproducibility). Cells were grown overnight in 10 mL LB medium supplemented with kanamycin (50 μg/mL) for clones amplification (Figure 1A). We then spread 150 μL of the libraries on agar plates containing kanamycin and different classes of antibiotics at concentrations that were inhibiting the wild-type strain [100 μg/mL ampicillin, 25 μg/mL cefuroxime, 40 μg/mL amoxicillin + 20 μg/mL clavulanic acid, 10 μg/mL chloramphenicol (Cm), 23.75 μg/mL sulfamethoxazole + 1.25 μg/mL trimethoprim (SXT), 600 μg/mL sulfamethoxazole and 10 μg/mL tetracycline] and incubated for 24 h at 37°C with 5% CO_2_. Similar concentrations for ampicillin and tetracycline have been used previously (Luo and Farrand, 1999). We aimed at using the same classes of antibiotics for functional metagenomics as used for the treatment of the infants (Supplementary Table 1). Up to 50 clones non-susceptible against a specific antimicrobial agent were picked, pooled in liquid LB supplemented with the appropriate antibiotics and grown for 6 h at 37°C (to obtain a representative clone population). Plasmids were extracted using (Promega, A1460, elution in 50 μL nuclease free water) from 1 mL of clones culture and inserts were amplified with M13 primers (Forward: 5′-GTAAAACGACGGCCAG-3′, Reverse: 5′-CAGGAAACAGCTATGAC-3′, final concentration 0.2 μM each) using 0.2 U iProof polymerase (Bio-Rad, #1725301) with final concentration of 200 μM each dNTP for a total reaction of 25 μL. PCR cycling protocol was: Denaturation for 2 min at 98°C, 30 cycles of 30 s at 98°C, 30 s at 51°C for annealing, 5 min at 72°C and a final amplification for 7 min in at 72°C. We then purified the PCR products (Promega, A9281, elution in 30 μL Elution Buffer).

### DNA Library Preparation and Nanopore Sequencing

We used nanopore MinION sequencing from Oxford Nanopore Technology (ONT) which is a technology currently under development. We used the 96 PCR Barcoding Kit (ONT, EXP-PBC096) and ligation sequencing kits for two directions (2D) (SQK-NSK007) or 1D (SQK-LSK108) for flow cells version R9 or R9.4 version to prepare the libraries, respectively. We pooled purified PCR products in a way so that a specific non-susceptible phenotype from a given sample was identified by a unique barcode per sequencing run. Using 200 μL tubes, 22.5 μL of purified and pooled PCR products were end-repaired and A-tailed using the Next Ultra II End Repair/dA-Tailing Module (NEB, E7546L) following manufacturer’s instructions for a final reaction volume of 30 μL and incubated for 30 min at 20°C and then 30 min at 65°C. End-repaired products were purified using 15 μL of AMPure XP beads (Beckman Coulter Inc., A63881) at room temperature for 5 min. Beads were pelleted on a 96-wells magnet and washed twice with 150 μL fresh 70% ethanol. DNA was eluted in 30 μL elution buffer (EB; Qiagen) before ligation with 20 μL of Barcode Adapter (ONT, EXP-PBC096), 10 μL Blunt/TA ligase Master mix (NEB, M0367L), 9 μL 10x T4 buffer (NEB, B02020S) and nuclease free water up to a final volume of 100 μL. Enzyme was heat inactivated at 70°C for 15 min. We then performed the ‘barcoding’ PCR (ONT, EXP-PBC096) including 5 μL of adapted DNA in a total reaction volume of 50 μL. We verified the amplification of the barcoded products by loading 2 μL of DNA before and after PCR on a 1% agarose gel. Finally we pooled 8 μL of barcoded PCR products from each sample and purified the DNA with 500 μL AMPure XP beads and incubated at room temperature for 5 min with rotation. After having washed twice with 500 μL fresh 70% ethanol, the DNA library was eluted in 45 μL EB. Libraries were prepared following manufacturer’s instructions (ONT, SQK-NSK007 or SQK-LSK108) with the following increased incubation times: 30 min at 20°C and 30 min at 65°C for the end-repair/A-tailing reaction and 30 min for ligation. We loaded the libraries following provider’s instructions on flow cells version R9.0 or R9.4 for 2D or 1D sequencing, respectively.

### Read Analysis

After 2D sequencing, we ran Metrichor^1^ (from ONT) for basecalling with subsequent demultiplexing of the barcoded 2D reads. Reads from 1D sequencing were basecalled and barcodes demultiplexed using Albacore (1.0.4). In case less than 1000 reads were obtained for a specific barcode, we repeated the sequencing of the corresponding inserts. We used poretools (Loman and Quinlan, 2014) to convert fast5 to fastq files. For each barcode, reads were assembled into contigs using Canu (1.4) (Koren et al., 2017). In order to obtain contigs longer than 500 bp and keep the possible variety of inserts, we used the following parameters for the assembly: -genomeSize = 0.022 m (An estimate of the size of the genome; in our case it is an estimate of the insert) -corMinCoverage = 5 (Limit read correction to regions with at least this minimum coverage) errorRate = 0.035 -contigFilter = “5 500 1.0 1.0 2” (min number of reads assembled, minimum length of 500 bp, single read span fraction of the contig, lower coverage span fraction, lowest coverage depth). In the 45 cases (20.7%) where Canu assembly failed because of a low number of reads, we increased the parameter for estimated error rate to 0.045 and in case of second failure (11 cases, 5%) we repeated the sequencing of those inserts. Primers and vector sequences were removed to obtain the final insert length. For 15 samples (6.7%), we observed that two identical PCR products were ligated together during the library preparation resulting in contigs containing repetitive sequences. We identified genes conferring resistance toward antibiotics according to the best hits obtained from BLAST against NCBI nucleotide (nt) database (online tool, December 2016–July 2017). The sequences were also compared online with The Comprehensive Antibiotic Resistance Database (CARD, July 2017, loose option) (Jia et al., 2017), Antibiotic Resistance Genes database (ARDB) (Liu and Pop, 2009) and ResFinder databases (Zankari et al., 2012). Sequences with less than 85% identity over 90% of sequence to known antibiotic resistance genes were classified as non-described, potentially novel proteins.

### Mobile Genetic Elements Detection and Phyla Analysis

To investigate the presence of mobiles genetic elements (MGE), we compared our sequences with the ICEberg (Inserted and Conjugative elements) and ISFinder databases (Sternberg, 1881; Bi et al., 2012). We also used RAIphy with the “iterative refinement” option to predict the taxonomic origin of our functionally selected DNA fragments (Pedersen and Henrichsen, 1983). As previously described, this composition-based classifier determines 7-mer profiles and compares those profiles to those of RefSeq genomes to accurately relate DNA fragments with a phylum source (Forsberg et al., 2014).

### Phylogenetic Tree of Amino Acid Sequences

Predicted protein sequences were obtained using ORFfinder (NCBI) (≥50 aa in length), aligned with MUSCLE (software MEGA, 5.2.1) and approximately-maximum-likelihood phylogenetic trees were built using FastTree^2^ (July 2017). The β-lactamases were categorized according to Ambler classification (Bush and Jacoby, 2010). Predicted protein sequences with less than 85% identity over 90% of sequence to known β-lactamases were classified as non-described proteins.

### Extended-Spectrum β-Lactamase (ESBL) Screening

In addition, clones with an inserted class A or C β-lactamase and non-described proteins were tested for extended-spectrum β-lactamase (ESBL) phenotype using the double-disk synergism test (DDST) on cation-adjusted Mueller-Hinton plates, with and without cloxacillin (250 μg/mL) for the discrimination of AmpC β-lactamases (Al Naiemi et al., 2009).

### Protein Network

All identified genes coding for resistance proteins were listed according to their non-susceptibility and grouped into the main classes of antibiotics: β-lactamases (with Ambler classification), dihydrofolate reductases (DHFR, folA or dfrG), thymidylate synthase, ribosomal protection protein, acetyltransferase, transporters and efflux pumps (ABC, MSF, or others) and non-described proteins. A connectivity matrix was generated with yEd Graph Editor (3.17.1) and a protein network built using Cytoscape (3.5.1). Size of the nodes represented the frequency of identification of a specific gene.

### Statistical Analysis

Samples from infants were grouped as (1) AB-naïve (without any AB history), (2) collected during an AB treatment, (3) collected after a single AB treatment, or (4) collected after more than one AB therapy. We then applied χ^2^ tests for 2 × 2 or 2 × 4 contingency tables to test the associations of the tested antibiotics with AB treatment history of the CF infants. Operational Taxonomic Units (OTU) for all samples were obtained from a previous study on the exact same samples (Mika et al., 2016). We calculated the beta-diversity using the *vegan* package in R with the *vegdist* function (Oksanen, 2013) using abundance- or binary-based Jaccard dissimilarity indices. For these analyses, infants who only provided a single sample for resistome analysis were excluded (*n* = 2). Plots illustrating antimicrobial susceptibility or non-susceptibility were generated using the *metaMDS* function with non-metric multidimensional scaling (nMDS) as ordination method.

The sequence reads from functional resistome experiments were submitted to the European Nucleotide Archive (accession number: PRJEB24475).

### Metagenomic Sequencing for Validation

To remove human reads from the metagenomic dataset, the Illumina reads were mapped against the human genome (GRCh38 with Decoy) using Bowtie 2 (Langmead and Salzberg, 2012) (Version 2.3.0). The fractions of human reads were 95.08, 97.64, and 85.62% for the samples CF1, CF2 and CF3, respectively. The remaining non-human reads (SRA-Accession: PRJEB30595) were mapped against the resistance gene databases ResFinder (Zankari et al., 2012) and MEGARes (Lakin et al., 2017) using Bowtie 2 (Langmead and Salzberg, 2012) (Version 2.3.0). All genes that were covered by at least one read were identified and analyzed using SAMtools (Li et al., 2009) (version 1.3).

## Results

### Study Population and Description of Non-susceptible Phenotypes Within Nasal Swabs

We performed functional metagenomic analyses (Figure 1A) of 130 nasal swabs from 26 infants with CF for this study. The sample collection included 72 swabs (55.3%) received during or following AB treatment (Figure 1B). Patient characteristics are outlined in the online supplement (Supplementary Table 1). We generated expression libraries for all samples (*n* = 130), and resulting transformants were then screened on solid agar media amended with a range of different classes of antibiotics (Figure 1A). We aimed at including the same classes of antibiotics as used for the treatment of the infants with CF (Supplementary Table 1). We obtained one or more non-susceptible phenotypes against the tested AB for 68 (52.3%) samples [mean: 1 non-susceptible phenotype, standard deviation (SD): 1.5]. Among all non-susceptibilities, 52 (40%) and 41 (32%) samples were β-lactams-non-susceptible and non-susceptible against folic acid synthesis inhibitors, respectively. We also observed 10 (8%) and 13 (10%) samples being tetracycline- and chloramphenicol- (Cm) non-susceptible, respectively.

### Catalog of Antimicrobial Resistance Genes (ARG) and Mobile Elements of Functional Metagenomic Libraries

For subsequent Nanopore MinION sequencing, we barcoded the libraries and then performed four sequencing runs using 2D (1 run) or 1D (3 runs) kits (Supplementary Tables 2–4 and Supplementary Figures 1–4). In total, we used up to 65 different barcodes per sequencing run (Supplementary Table 2) and the total number of clones are illustrated in Supplementary Tables 3, 4. We sequenced up to 50 clones per sample (i.e., 50 colonies were picked for sequencing if ≥50 colonies were counted, all colonies were picked if ≤ 50 colonies). Overall, we obtained a mean of 16,004 reads for each barcode (*n* = 217). We then used a total number of 65,394 good quality reads for the subsequent assemblies (Supplementary Tables 2–4 and Supplementary Figures 1–4). Average lengths of DNA inserts were 3.0 kb (SD: 1.4 kb, Supplementary Figures 5A,B). In total, we assembled 213 contigs > 500 bp and identified 171 resistance genes.

**FIGURE 2:**
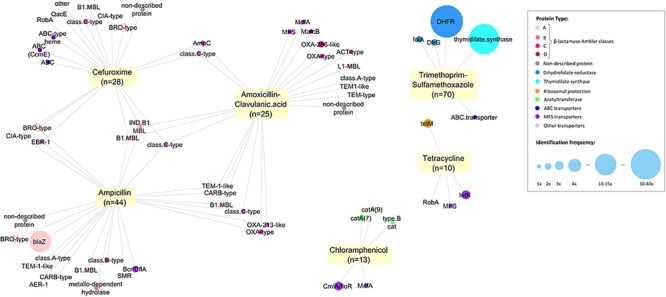
Network of all proteins identified conferring antitiotic resistance. We identified 171 resistance proteins and grouped them according to the main classes of antibiotics observed: β-lactamases (with Ambler classification), dihydrofolate reductases (DHFR, folA or dfrG), thymidylate synthase, ribosomal protection protein, acetyltransferase, transporters and efflux pumps (ABC, MSF or others) and non-described proteins. The respective non-susceptible phenotypes were recorded and a network was built using Cytoscape (3.5.1). Each node represents a protein type identified and is connected to the antibiotics for which it presented non-susceptibility. Size of the node represents the frequency of identification.

For all tested ABs, we identified efflux systems as the mechanism conferring resistance of which ABC and MSF transporters were the most common (4.6 and 7.7% of 130 samples respectively) (Figure 2). The resistance genes *CmlA* (chloramphenicol efflux pump) and *MdfA* (multidrug transporter) were the most frequent proteins observed conferring Cm-non-susceptibility. For tetracycline-non-susceptibility, *tet*(K) was the most frequently identified protein (2.3%). Dihydrofolate reductase (26.9%) and thymidylate synthase enzymes (10.7%) were the most common proteins identified for folic acid synthesis inhibitors (Figure 2).

**FIGURE 3:**
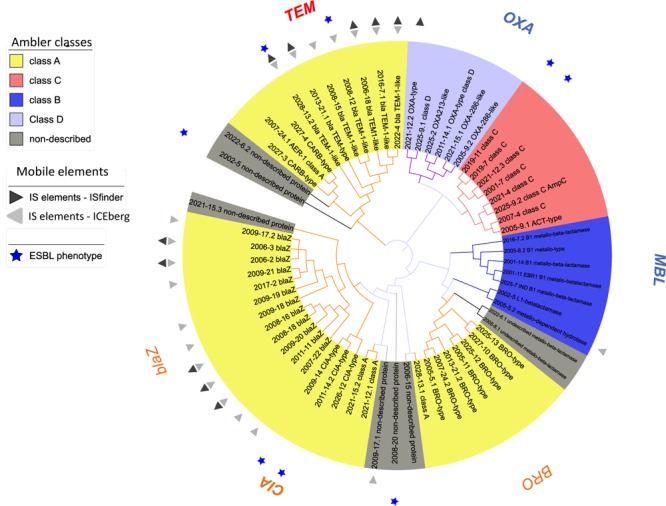
Phylogenetic tree of predicted β-lactamases identified. Predicted protein sequences were obtained using ORFfinder (NCBI) (≥50 amino acids in length) using the genes received from functional metagenomics sequencing. β-lactamases (*n* = 64) were assigned to an Ambler class according the best hit from blast against NCBI. Sequences with less than 85% identity over 90% of sequence to known β-lactamase were classified as non-described proteins. All sequences were aligned with MUSCLE (software MEGA, 5.2.1) and an approximately-maximum-likelihood phylogenetic tree was built using FastTree (http://www.microbesonline.org/fasttree/, July 2017).

Overall, β-lactams-non-susceptibility was most frequently identified including 64 different predicted amino acid sequences recovered coding for β-lactamases (Figure 3). We obtained enzymes from all Ambler classes. We observed eight predicted amino acid sequences with less than 85% identity over 90% of sequence to any known β-lactamase and classified them as non-described proteins. All non-described proteins conferred non-susceptibility against all three β-lactams tested (ampicillin, cefuroxime or amoxicillin-clavulanic acid). We also observed ESBL phenotypes for eight recovered proteins (Figure 3 and Table 1). Overall, mobile elements were discovered for 21 of 64 (32.9%) β-lactamases (Figure 3). The recovery of mobile elements was independent of the lengths of the contigs (Supplementary Figure 5A). However, a sequence length dependency was received if only the β-lactamases were included (*p* < 0.05; Supplementary Figure 5B). As for non-β-lactamases, MGE were also received for some other genes, including *tetM*.

**Table 1:** All proteins conferring β-lactam non-susceptibilities.

		Non-susceptible phenotype				
Ambler classes for β-lactamases		N_Total_	Ampicillin	Amoxicillin-clavulanic acid	Cefuroxime	ESBL-like
A	AER-1	1	1	0	0	
	TEM-1-like	6	5	4	2	2
	TEM-type	2	0	2	0	
	blaZ	12	12	0	0	
	BRO-type	7	6	0	4	
	CARB-type	2	2	1	0	
	CIA-type	3	1	0	3	2
	Class A-type	2	1	1	0	
B	B1-MBL	5	4	2	2	
	EBR-1 B1-MBL	1	1	0	1	
	IND B1-MBL	1	1	1	1	
	L1- MBL	1	0	1	0	
	Metallo-dependent hydrolase	1	1	0	0	
C	ACT-type	1	0	1	0	
	AmpC	1	0	1	1	
	Class C-type	6	2	3	5	2
D	OXA-213-like	1	1	1	0	
	OXA-286-like	2	0	2	0	
	OXA-type	2	1	2	0	
	Class D-type	1	1	0	0	
Unknown	non-described protein	6	1	2	3	2

**Transporter/efflux pump**		**N_Total_**	**Ampicillin**	**Amoxicillin-clavulanic acid**	**Cefuroxime**	**ESBL**

	ABC transporter	6	0	1	5	
	MFS transporter	3	1	2	0	
	Other transporters	5	2	0	3	

*MBL, metallo- β-lactamases; ESBL, extended-spectrum β-lactamases.*

### Accuracy of Annotation of Nanopore Sequencing Reads

We also investigated how the error rate within reads produced by Nanopore sequencing could affect the ARGs detection and identification (annotation). We generated mutant sequences from the original contigs with different mutation rates (point mutations) and challenged them against CARD database (Supplementary Material). The presence of ARGs was detected in more than 90% of the cases even with 3% of mutations introduced into the sequences, with the exception of the sequences coding for BRO-type β-lactamases (Supplementary Figure 6), for which the detection decreased rapidly with 1% of mutations. The identification of β-lactamase gene variant was affected by increasing mutation rates and introducing point mutations of 3% introduced already a misclassification of PDC-7 (AmpC-type, ESBL) in CMY-11 (class C β-lactamase).

### Resistome Association With AB Treatment and Bacterial (Microbiota) Composition

**FIGURE 4:**
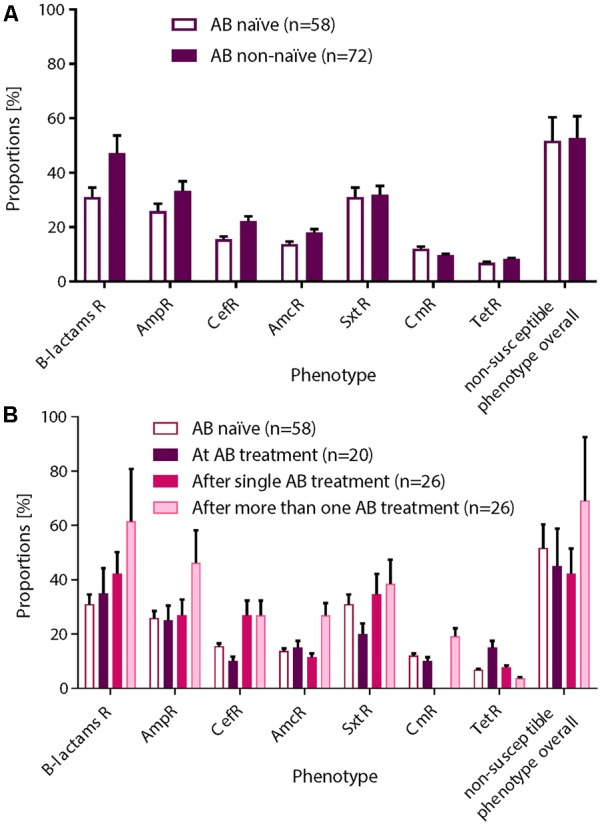
Non-susceptible phenotypes were not associated with AB treatment history. After functional metagenomics analysis of all samples (*n* = 130), proportions of non-susceptible phenotypes observed for all AB tested were calculated according to the AB status of the patient: **(A)** AB naïve vs. non-naïve or **(B)** AB naïve (group 0), collected during an AB treatment (group 1), collected after a single AB treatment (group 2), or collected after more than one AB therapy (group 3). A χ^2^ test for a 2 × 2 or 2 × 4 contingency tables was applied for statistical significance. **(A)** Non-susceptible phenotypes were more frequently recovered from patient with AB history. **(B)** We observed an increasing trend for β-lactams resistance in patients who received one or more AB therapies. Standard deviations are indicated.

**FIGURE 5:**
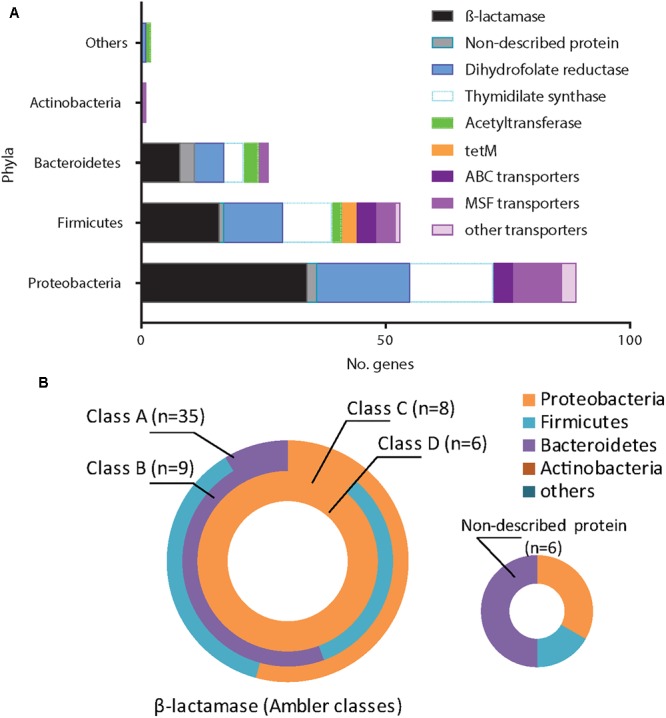
Antimicrobial resistance genes (ARG) and the related phyla. We related all identified ARG (*n* = 171) to the closest bacterial phyla using RAIphy on the contigs sequences obtained. Protein sequences were grouped according the main following families: β-lactamases (with Ambler classification), dihydrofolate reductases, thymidylate synthase, ribosomal protection protein, acetyltransferase, transporters/efflux pumps (ABC, MSF or others) and non-described proteins. We reveal the Proteobacteria, Firmicutes, Bacteroidetes, Actinobacteria, while other phyla are grouped into the “others.” **(A)** The majority of ARG came from Proteobacteria and Firmicutes. **(B)** Classes A and B β-lactamases were related to Proteobacteria, Firmicutes or Bacteroidetes while class C and D were exclusively related to Proteobacteria.

We next investigated the association of the resistome with the AB treatment of the CF patients (Figure 4A,B). There were no significant differences in the proportions of non-susceptible phenotypes between patients without an AB history, during AB treatment or after therapy (Figure 4A,B). However, we observed a non-significant trend for higher β-lactams-non-susceptibility in infants with a history of one or more AB therapies (Figure 4B). We then predicted the possible taxonomic source (phylum) of each functionally selected resistance contig by using RAIphy. Proteobacteria and Firmicutes were the most prevalent predicted phyla (Figure 5A), and β-lactamases showed the strongest, and most predominant relationships with predicted bacterial phyla. The β-lactamases from class A and B were enriched within Proteobacteria, Firmicutes, and Bacteroidetes, but class C and class D β-lactamases were exclusively predicted for Proteobacteria (Figure 5B).

**FIGURE 6:**
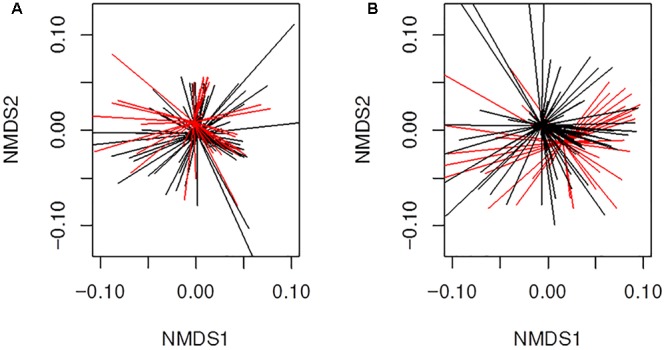
Beta-diversity analyses of all samples (*n* = 130) from infants with CF for ampicillin resistance using non-metric multidimensional scaling (nMDS). Illustrated are **(A)** results for abundance based distance matrix (permutational multivariate ANOVA *P* = 0.04) and **(B)** binary based distance matrix (permutational multivariate ANOVA *P* = 0.001). Ampicillin-non-susceptible samples were more similar to each other than compared with susceptible sample. Red; Sample is ampicillin-non-susceptible. Black; Sample is susceptible toward ampicillin. Ordispider plots are illustrated which combine items to their centroid.

We also analyzed more closely if the β-lactams-non-susceptibility was associated with significantly different microbial community clusters derived from previously received 16S rRNA gene data (Mika et al., 2016). Therefore, we created nMDS plots based on the abundance and binary based distance matrices of the operational taxonomic units (97% sequence identity) and performed PERMANOVA statistics with β-lactams-non-susceptibilities as factors. There was a significant separation for both, abundance (Figure 6A; *P* = 0.04) and binary-based analyses for ampicillin (Figure 6B; *P* = 0.001). The differences were less apparent but still significant for cefuroxime and amoxicillin-clavulanic acid.

### Comparing Functional Resistome With Culture-Independent Shotgun Metagenomics Sequencing

**FIGURE 7:**
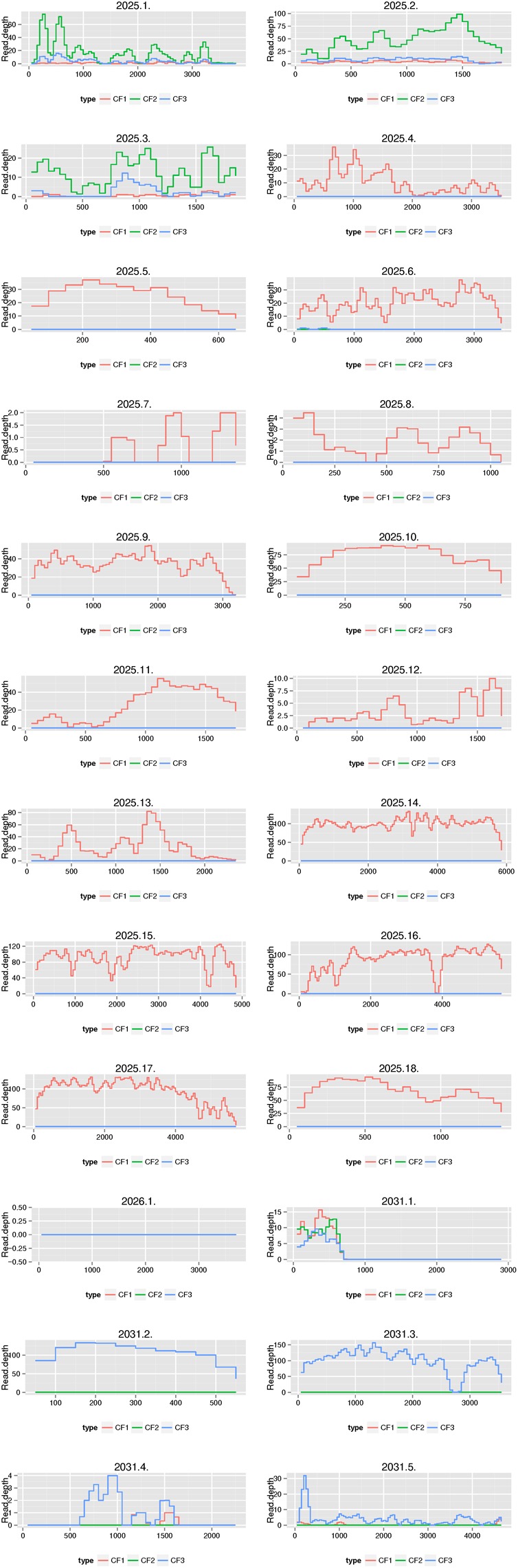
Comparison of the functional resistome with shotgun metagenomics sequencing for three samples. Functional resistome analyses revealed 18 genes for sample ID CF1 (2025.1–2025.18), 1 gene for CF2 (2026.1) and 5 genes for CF3 (2031.1-2031.5). The plots illustrate the sequence coverage from the shotgun sequencing for the genes identified by functional genomics. We recovered reads from shotgun metagenomics sequencing for 18/18 (for ID 2025), 0/1 (for ID 2026), and 5/5 (for ID 2031) resistance genes. Certain resistance genes (e.g., 2025.1, 2025.2, and 2025.3) were found in multiple samples [indicated as CF1 (ID 2025), CF2 (ID 2026) and CF3 (ID 2031)].

We then compared the outcomes of the functional resistome with culture-independent shotgun metagenomics sequencing for three samples. Based on our functional resistome approach, the three samples showed several phenotypic resistances (Sample ID CF1 was resistant for ampicillin, amoxicillin clavulanic acid, cefuroxime sulfamethoxazole-trimethoprim, tetracycline and chloramphenicol; Sample ID CF2 was resistant to cefuroxime and Sample ID CF3 was resistant for sulfamethoxazole-trimethoprim, chloramphenicol and tetracycline) for which 18, 1, and 5 resistance genes were found for CF1, CF2, and CF3 respectively. The numbers of received sequences were 27,038,599 (for CF1), 32,276,677 (for CF2) and 36,395,636 (for CF3) and the sequence coverages from the shotgun sequencing for the genes identified by functional genomics are shown in Figure 7. We recovered reads from culture-independent shotgun metagenomics sequencing for 18/18 (for ID Cf1), 0/1 (for ID CF2) and 5/5 (for ID CF3) resistance genes. However, for two genes (2031.1 and 2031.4), the read coverage was quite low. Certain resistance genes (e.g., 2025.1, 2025.2 and 2025.3; all BRO-type Beta-Lactamases) were found in multiple samples [indicated as CF1 (ID 2025), CF2 (ID 2026), and CF3 (ID 2031)]. Additional resistance genes were identified using Resfinder for the shotgun metagenomics reads (Supplementary Tables 5–7). This included hits for antibiotics which were not (Lincosamide and Fosfomycin) or could not be tested by the functional resistome approach using *E. coli* as a host with kanamycine resistance plasmids (macrolides and aminoglycosides). However, the read coverage was generally low as compared to the opposite search as outlined above.

## Discussion

It has been postulated that CF patients have complex lung microbiota within which there are many genetic exchanges occurring by horizontal gene transfer. This promotes both, the emergence and selection of multidrug-resistant bacteria that may spread in this population and the evolution of the microbial community that is specific to the disease (Rolain et al., 2011). In this study, we analyzed the nasal resistome of infants with CF during the first year of life using a functional metagenomics protocol. We report that multiple antibiotic non-susceptibilities including ESBLs are present within the first months of life even if the infants have not yet been exposed to antibiotic treatment. This indicates that antibiotic treatment may not play a major role in the development of the resistome in the first year of life. In addition, we show that microbiota composition is associated with specific non-susceptible phenotypes. The latter makes it challenging to differentiate between changes in the carriage of resistance genes due do direct selective antibiotic pressure versus changes in resistance genes carriage, because of shifts in the relative abundance of the bacterial populations that encode them which has already been noted in an earlier study (Taylor et al., 2018).

The functional resistome approach enables detecting antibiotic resistance phenotypes and resistances that have not yet been described. This cannot be achieved by the shotgun sequencing methods which have recently been used for the investigation of the resistome in CF (Lim et al., 2014; Bacci et al., 2017; Feigelman et al., 2017). However, as compared to functional metagenomics, shotgun sequencing methods are probably more sensitive because the used *E. coli* model may present a poor system for the discovery of resistance genes that originated from Gram-positive organisms (Crofts et al., 2017). We therefore compared functional resistome analyses with shotgun sequencing results for three samples. We indeed revealed resistance genes for antibiotics (e.g., macrolides and aminoglycosides) which were not detected by our functional resistome approach. We hypothesize that both methods have advantages and disadvantages and may be considered in the future for resistome analyses in CF.

Using functional metagenomics, we generally detected a large number of genes coding for β-lactam resistances of which some of them were well known and their detection was expected. This include the genes *bla*_TEM-1_ and *bla*_BRO-type_ which are very frequently present in *Haemophilus influenzae* and *Moraxella catarrhalis* isolates, respectively (Farrell et al., 2005; Hsu et al., 2012). Similarly, *blaZ* is often detected in penicillin-non-susceptible *Staphylococcus aureus* and coagulase-negative staphylococci (Olsen et al., 2006). Interestingly, we found that the resistome also includes the presence of some ESBLs, which are clinically relevant as they could severely affect potential treatment outcomes. It was recently speculated that the production of ESBL by commensal bacteria of the CF respiratory microbiota might have a potential indirect pathogenic role (Sherrard et al., 2016). However, even the observation of some ESBLs in our study may be unsurprising as, the *bla*_CIA-type_ gene encodes an ESBL that has been found within *Chryseobacterium* spp. (Matsumoto et al., 2012). Multidrug resistance in Chryseobacteria has also been recently described in a CF patient (Sharma et al., 2015). However we were able to detect two ESBLs with mobile elements and a potentially new ESBL, though we could not accurately trace the bacterial species, as our chosen method is limited for this purpose.

We also investigated non-β-lactam-non-susceptibilities in our study (Supplementary Table 1). Tetracycline-non-susceptibilities were rare with *tet*(K) and *tet*(M) within Firmicutes the most frequently detected ARGs. The *tet*(M) gene has been described as being highly mobilizable and includes the detection within Proteobacteria (Endimiani et al., 2017). As for trimethoprim-sulfamethoxazole, selections containing trimethoprim and D-Cycloserine predominantly recovered dihydrofolate reductases, D-alanine-D-alanine ligases, and thymidylate synthases, which are well-known enzymes conferring resistance and have been frequently described in other habitats (Forsberg et al., 2014). Finally It has been shown that bacteria, such as *Streptococcus pneumoniae*, can grow in medium supplemented with Cm when non-susceptible bacteria expressing Cm acetyltransferase (cat) are present, suggesting the relevance for functional metagenomics investigation of the resistome for Cm (Yurtsev et al., 2016).

Based on our results, we hypothesize that the infant nasal microbiota and resistome does not yet reach final maturation within the first year of life. A very recent study hypothesized, that the majority of CF subjects older than four harbor a pathogen dominated airway microbiome, compared to younger patients with an oral-like airway microbiome (Muhlebach et al., 2018). As for healthy children, it has been speculated that the maturation of the microbiota composition is achieved after children reach 2 years of age (Stearns et al., 2015).

In our study, we used Nanopore MinION sequencing which has some distinct advantages. Nanopore reads are longer and typically capture the entire metagenomic inserts (van der Helm et al., 2017), including sequences of MGE and/or insertion sequences, which assures the definition and therefore enable a better characterization of the “mobilizable” resistome. In addition, the well-established barcoding protocols allow the parallel processing of several samples and decrease considerably sequencing cost. However, the error rates are still higher as compared to other sequencing technologies (Loman et al., 2015; Deschamps et al., 2016). This may be especially true in the case of 1D (as compared to earlier 2D) sequencing for which many raw reads need to be received to ascertain some high quality contigs after trimming and assembly (Loman et al., 2015). However, we investigated how mutations or error rate could potentially impact on the ARGs detection and found that the detection of ARGs was robust even with a high proportion of mutations. Furthermore, as Oxford Nanopore Technologies constantly make improvement on the accuracy and throughput of nanopore reads, the results obtained in this study are rather conservative and the approach is expected to improve qualitatively with time.

There are major strengths in our study. We used a functional metagenomics approach that directly targets antibiotic non-susceptibly rather than providing overall and unspecific genomic information. With this approach, well-known but also new resistance mechanisms can be more directly identified. Nanopore sequencing proved to be ideal for our study as the technique produces long reads which facilitate the capture of the entire metagenomic insert including, if present, mobile elements (van der Helm et al., 2017). In addition, we have included a large number of samples (*n* > 100) from a well-defined CF cohort of infants during their first year of life (Mika et al., 2016). For all samples, functional resistome and 16S rRNA gene sequence data were available. Our study also had some limitations. First, the sensitivity of functional metagenomics is limited as nasal swabs have generally a low density of bacteria and, therefore, whole functional resistome profiling is not able to come up with an exhaustive description of the antibiotic resistance gene repertoire. In addition, we had to perform prior multiple displacement amplification which is known to be error prone. We can also not guarantee that our libraries are comprehensive as it was difficult to estimate the library size which is normally a usual standard approach for functional resistome projects (Moore et al., 2013). Also, some resistance mechanisms may still remain undetected, such as those related to distinct single nucleotide polymorphisms (SNPs) within penicillin binding proteins of gram positive bacteria like *S. pneumoniae* (Engel et al., 2014). We exclusively used the *E. coli* model as we were primarily interested in the non-chromosomal, plasmid-mediated resistance mechanisms (hence long read sequencing), especially Beta-Lactamases. We indeed found two potential new ESBLs within mobile elements. However, a standard operating procedure for functional metagenomics using a gram positive bacterium rather than *E. coli* has to be additionally established in the near future.

While the functional resistome has the potential to influence clinical treatment, its implementation and relevance in the treatment of patients with CF have to be more clearly investigated in future studies using a larger group of children with CF. In addition, we need more samples and information concerning the ‘healthy’ resistome of the upper (including the nose) and lower respiratory tract.

## Conclusion

We characterized the functional resistome of the nose in infants with CF during the first year of life. We revealed the presence of a large number or ARG, even if the infants were not yet exposed to antibiotic treatment. Understanding the resistome could potentially optimize clinical decision-making in patients with CF but this needs to be further investigated in future studies using clinically relevant samples.

## Members of the SCILD Study Group

Jürg Barben, MD, St. Gallen; Juerg.Barben@kispisg.ch. Carmen Casaulta, MD, Bern; Carmen.Casaulta@insel.ch. Andreas Jung, MD, Zurich; andreas.jung@kispi.uzh.ch. Elisabeth Kieninger, MD, PhD, Bern; elisabeth.kieninger@insel.ch. Insa Korten, MD, Bern; Insa.Korten@insel.ch. Philipp Latzin, MD, PhD, Bern; Philipp.Latzin@insel.ch. Alexander Moeller, MD, Zurich; Alexander.Moeller@kispi.uzh.ch. Anne Mornand, MD, Geneva; Anne.Mornand@hcuge.ch. Gaudenz Hafen, MD, Lausanne; gaudenz.hafen@chuv.ch. Dominik Müller-Suter, MD, Aarau; Dominik.Mueller-Suter@ksa.ch. Nicolas Regamey, MD, Lucerne; nicolas.regamey@luks.ch. Isabelle Rochat MD, Lausanne; Isabelle.Rochat@chuv.ch. Florian Singer, MD, PhD, Bern; florian.singer@insel.ch. Renate Spinas, MD, Zurich; renate.spinas-haeller@kispi.uzh.ch. Daniel Trachsel, MD, Basel; daniel.trachsel@ukbb.ch. Sophie Yammine, MD, PhD, Bern; Sophie.Yammine@insel.ch. Maura Zanolari, MD, Bellinzona; m.zanolari@bluewin.ch.

## Author Contributions

AA conducted the vast majority of experiments. AA, PL, and MH designed the study and analyzed the data. AA, JK, IK, KR, DW, CC, PL, and MH collected and generated the data. AA, JK, AR, AE, PL, and MH advised on data analysis and manuscript writing. All authors read and approved the manuscript.

## Conflict of Interest Statement

MH has received a grant from the Swiss Lung Association Bern and from the Swiss National Science Foundation (No. 320030_159791). PL reports personal fees from Gilead, Novartis, Polyphor, Roche, Schwabe, Vertex, Vifor, and Zambon, all outside the submitted work. The remaining authors declare that the research was conducted in the absence of any commercial or financial relationships that could be construed as a potential conflict of interest.

## Footnotes

1 https://metrichor.com

2 http://www.microbesonline.org/fasttree
