# Supplementary material for: Nasal Resistome Development in Infants With Cystic Fibrosis in the First Year of Life
Source: Front Microbiol. 2019 Feb 26;10:212. doi: 10.3389/fmicb.2019.00212 (PMC6399209; doi:10.3389/fmicb.2019.00212)

**Supplementary table 1: Patients characteristics**

|  |  | CF genotype | |  | | |  |  | Antibiotic treatment 1 | | | Antibiotic treatment 2 | | | Antibiotic treatment 3 | | | Antibiotic treatment 4 | | | Antibiotic treatment 5 | | | Antibiotic treatment 6 | | |
| --- | --- | --- | --- | --- | --- | --- | --- | --- | --- | --- | --- | --- | --- | --- | --- | --- | --- | --- | --- | --- | --- | --- | --- | --- | --- | --- |
| ID | month of birth | 1 | 2 | | Samples per infant for 16S | Samples per infant Resistom | | Month of the year sampled for Resistome | Start* | End * | Antibiotic | Start* | End * | Antibiotic | Start* | End * | Antibiotic | Start* | End * | Antibiotic | Start* | End * | Antibiotic | Start* | End * | Antibiotic |
| 2000 | Feb | ΔF508 | ΔF508 | | 22 | 0 | |  | 17·7 | 19·7 | Amox/Clav | 27·3 | 29·3 | Amox/Clav |  |  |  |  |  |  |  |  |  |  |  |  |
| 2001 | Mar | ΔF508 | ΔF508 | | 16 | 5 | | 07/08/09/10/11 |  |  |  |  |  |  |  |  |  |  |  |  |  |  |  |  |  |  |
| 2002 | Apr | ΔF508 | ΔF508 | | 11 | 5 | | 06/07/09/11/12 | 15·1 | 16·6 | others | 26·6 | 28·6 | Cotrim |  |  |  |  |  |  |  |  |  |  |  |  |
| 2003 | Apr | ΔF508 | 621+1G>T | | 14 | 5 | | 06/09/09/11/04 | 47·6 | 48·6 | Amox/Clav |  |  |  |  |  |  |  |  |  |  |  |  |  |  |  |
| 2004 | May | ΔF508 | Q525X | | 17 | 1 | | 12 | 7·3 | 8·7 | Cefuroxim | 11·0 | 13·0 | Amox/Clav | 21·3 |  | Cotrim | 28·3 | 29·6 | Cotrim |  |  |  |  |  |  |
| 2005 | May | ΔF508 | G486E | | 18 | 8 | | 08/09/09/10/10/11/12/05 | 20·1 | 20·9 | others | 28·4 | 29·7 | Amox/Clav | 33·4 |  | Amox/Clav | 45·6 | 47·0 | Amox/Clav |  |  |  |  |  |  |
| 2006 | Jun | ΔF508 | ΔF508 | | 19 | 6 | | 08/09/12/01/03/05 | 6·0 | 8·3 | NA | 12·1 | 15·1 | Cotrim | 25·1 |  | Amox/Clav | 37·9 | 39·7 | Amox/Clav | 42·4 | 44·4 | Amox/Clav |  |  |  |
| 2007 | Sep | ΔF508 | ΔF508 | | 24 | 8 | | 12/05/06/07/07/08/08/09 | 6·4 | 8·3 | Amox/Clav | 39·4 | 41·4 | Amoxicillin | 48·7 |  | others |  |  |  |  |  |  |  |  |  |
| 2008 | Oct | ΔF508 | R347H | | 17 | 12 | | 11/12/12/01/01/03/04/05/06/06/07/8 | 4·9 | 6·4 | Amox/Clav | 13·0 | 14·6 | Cefuroxim | 36·6 |  | Amox/Clav | 49·1 | 50·4 | Amox/Clav |  |  |  |  |  |  |
| 2009 | Oct | ΔF508 | ΔF508 | | 20 | 10 | | 11/12/03/05/06/07/07/08/08/09 | 4·9 | 5·9 | Cefuroxim | 24·7 | 26·6 | Amox/Clav | 49·6 |  | Amox/Clav |  |  |  |  |  |  |  |  |  |
| 2010 | Dec | ΔF508 | ΔF508 | | 18 | 1 | | 06 |  |  |  |  |  |  |  |  |  |  |  |  |  |  |  |  |  |  |
| 2011 | Jan | ΔF508 | R1162L | | 15 | 3 | | 08/09/10 | 9·1 | 10·7 | NA | 45·6 | 47·6 | Amox/Clav |  |  |  |  |  |  |  |  |  |  |  |  |
| 2013 | Mar | ΔF508 | D1152H | | 22 | 4 | | 08/12/02/03 |  |  |  |  |  |  |  |  |  |  |  |  |  |  |  |  |  |  |
| 2014 | Apr | ΔF508 | 3905 ins T | | 14 | 2 | | 05/11 | 31·3 | 33·4 | Amox/Clav | 40·3 | 42·3 | Amox/Clav |  |  |  |  |  |  |  |  |  |  |  |  |
| 2015 | Mar | ΔF508 | R347H | | 16 | 0 | |  | 8·4 | 9·9 | NA | 40·0 | 41·3 | Amox/Clav |  |  |  |  |  |  |  |  |  |  |  |  |
| 2016 | May | ΔF508 | ΔF508 | | 17 | 1 | | 10 |  |  |  |  |  |  |  | 23·3 |  |  |  |  |  |  |  |  |  |  |
| 2017 | May | ΔF508 | R553X | | 16 | 7 | | 07/09/10/11/01/02/04 | 28·6 | 29·7 | Amox/Clav |  |  |  |  | 34·9 |  |  |  |  |  |  |  |  |  |  |
| 2019 | Aug | ΔF508 | ΔF508 | | 16 | 6 | | 11/12/02/03/05/07 | 25·3 | 27·3 | Amox/Clav | 28·4 | 30·4 | Amox/Clav | 31·6 | 27·1 | Cotrim | 39·4 | 44·1 | Cotrim | 45·1 | 48·0 | Amox/Clav | 49·6 | 51·6 | Cef |
| 2020 | Nov | ΔF508 | N 1303K | | 12 | 0 | |  | 25·4 | 26·9 | Amoxicillin |  |  |  |  | 50·0 |  |  |  |  |  |  |  |  |  |  |
| 2021 | Decr | ΔF508 | ΔF508 | | 13 | 4 | | 05/10/10/11 | 28·1 | 30·1 | Cotrim | 35·3 | 38·3 | others |  | 38·4 |  |  |  |  |  |  |  |  |  |  |
| 2022 | Jan | ΔF508 | R347H | | 12 | 5 | | 05/05/06/07/10 | 25·3 | 26·3 | Amox/Clav |  |  |  |  | 50·9 |  |  |  |  |  |  |  |  |  |  |
| 2023 | Jan | ΔF508 | ΔF508 | | 12 | 0 | |  | 0·1 | 0·9 | others | 4·0 | 4·7 | others | 42·7 |  | Amoxicillin |  |  |  |  |  |  |  |  |  |
| 2024 | Mar | R553X | W1282X | | 4 | 1 | | 10 | 28·4 | NA | Amox/Clav | 39·4 | 41·0 | others |  |  |  |  |  |  |  |  |  |  |  |  |
| 2025 | Apr | ΔF508 | ΔF508 | | 16 | 8 | | 07/08/10/10/01/01/02/03 |  |  |  |  |  |  |  |  |  |  |  |  |  |  |  |  |  |  |
| 2026 | Jun | R1066H | 3905insT | | 17 | 9 | | 09/10/11/01/01/02/03/05/06 | 27·4 | 28·6 | Amoxicillin | 42·6 | 44·6 | others | 47·1 | 48·6 | Amoxicillin |  |  |  |  |  |  |  |  |  |
| 2027 | Aug | ΔF508 | 711+5G>A | | 12 | 4 | | 11/12/05/06 | 25·4 | 26·7 | Amoxicillin | 44·9 | 46·9 | Amox/Clav |  |  |  |  |  |  |  |  |  |  |  |  |
| 2028 | Nov | ΔF508 | ΔF508 | | 11 | 1 | | 07 | 41·3 | 42·7 | Amox/Clav | 44·6 | 45·9 | Amoxicillin |  |  |  |  |  |  |  |  |  |  |  |  |
| 2029 | Mar | ΔF508 | R553X | | 13 | 1 | | 10 |  |  |  |  |  |  |  |  |  |  |  |  |  |  |  |  |  |  |
| 2030 | Apr | ΔF508 | 2789+5G-A | | 15 | 2 | | 06/07 | 43·0 | 44·9 | Amox/Clav |  |  |  |  |  |  |  |  |  |  |  |  |  |  |  |
| 2031 | May | ΔF508 | 1717-1G>A | | 12 | 11 | | 07/07/08/08/09/09/10/10/11/12/12 | 26·7 | 28·1 | Amox/Clav | 37·3 | 37·4 | NA | 37·6 | NA | Cefuroxim | 44·3 | 45·3 | Cefuroxim | 48·7 | 49·7 | Cefuroxim |  |  |  |

*Weeks after birth

Supplementary table 2: Number of reads per sequencing run, before and after assembly

| sequencing | Mode of sequencing | Entries after demultiplexing [reads] | Number of reads per barcode (before assembly) [mean] | [95% CI] | Mean reads length [bp] | | Number of barcode used | Entries used for assemblies [reads] | Mean length [bp] |
| --- | --- | --- | --- | --- | --- | --- | --- | --- | --- |
| run 1 | 2D | 76’352 | 1’441 | 357 | 1502.6 | | 53 | 40’132 | 2’637 |
| run 2 | 1D | 1’582’934 | 16’985 | 2’299 | 1576.3 | | 49 | 7’191 | 3’400 |
| run 3 | 1D | 724’172 | 8’813 | 4’312 | 1132.1 | | 50 | 11’921 | 3’960 |
| run 4 | 1D | 1’089’343 | 15’579 | 4’062 | 647.3 | | 65 | 6’150 | 2’082 |
| all runs | 1D and 2D | 3’472’801 | 16’004 | 2’982 | 1190.7 | 217 | | 65’394 | 3’061 |

bp= base pairs; CI confidence interval

Supplementary table 3: number of clones, reads, contigs and unique genes identified per sample and β–lactams-non-susceptibility

|  |  | | | | β-lactams-non-susceptibility | | | |  | | | |
| --- | --- | --- | --- | --- | --- | --- | --- | --- | --- | --- | --- | --- |
| Antibiotic | **Ampicillin** | | | | **Cefuroxime** | | | | **Amoxi-Clav** | | | |
| Sample ID | **clones** | **reads** | **contig** | **unique gene sequences** | **clones** | **reads** | **contig** | **unique gene sequences** | **clones** | **reads** | **contig** | **unique gene sequences** |
| 2001-11 | 50 | 158 | 1 | 1 | 50 | 170 | 2 | 1 | 0 | 0 | 0 | 0 |
| 2001-12 | 0 | 0 | 0 | 0 | 0 | 0 | 0 | 0 | 0 | 0 | 0 | 0 |
| 2001-14 | 30 | 104 | 1 | 1 | 30 | 204 | 3 | 1 | 20 | 202 | 1 | 1 |
| 2001-7 | 0 | 0 | 0 | 0 | 65 | 33 | 6 | 2 | 0 | 0 | 0 | 0 |
| 2001-8 | 0 | 0 | 0 | 0 | 0 | 0 | 0 | 0 | 0 | 0 | 0 | 0 |
| 2002-10 | 0 | 0 | 0 | 0 | 0 | 0 | 0 | 0 | 0 | 0 | 0 | 0 |
| 2002-5 | 175 | 4 | 1 | 1 | 0 | 0 | 0 | 0 | 30 | 150 | 1 | 1 |
| 2003-7 | 0 | 0 | 0 | 0 | 0 | 0 | 0 | 0 | 0 | 0 | 0 | 0 |
| 2003-8 | 0 | 0 | 0 | 0 | 0 | 0 | 0 | 0 | 0 | 0 | 0 | 0 |
| 2004-13 | 0 | 0 | 0 | 0 | 0 | 0 | 0 | 0 | 0 | 0 | 0 | 0 |
| 2005-11 | 300 | 0/140* | 0/1* | 0/1* | 5 | 8 | 1 | 1 | 0 | 0 | 0 | 0 |
| 2005-4 | 0 | 0 | 0 | 0 | 0 | 0 | 0 | 0 | 0 | 0 | 0 | 0 |
| 2005-5 | 200 | 110 | 3 | 3 | 0 | 0 | 0 | 0 | 0 | 0 | 0 | 0 |
| 2005-6 | 0 | 0 | 0 | 0 | 0 | 0 | 0 | 0 | 0 | 0 | 0 | 0 |
| 2005-8 | 150 | 896 | 3 | 2 | 0 | 0 | 0 | 0 | 50 | 157 | 2 | 1 |
| 2005-9 | 0 | 0 | 0 | 0 | 0 | 0 | 0 | 0 | 3 | 168 | 3 | 2 |
| 2006-15 | 0 | 0 | 0 | 0 | 0 | 0 | 0 | 0 | 3 | 97 | 1 | 1 |
| 2006-18 | 0 | 0 | 0 | 0 | 0 | 0 | 0 | 0 | 8 | 33 | 1 | 1 |
| 2006-2 | 200 | 576 | 1 | 1 | 0 | 0 | 0 | 0 | 0 | 0 | 0 | 0 |
| 2006-3 | 300 | 13 | 1 | 1 | 0 | 0 | 0 | 0 | 0 | 0 | 0 | 0 |
| 2007-19 | 0 | 0 | 0 | 0 | 0 | 0 | 0 | 0 | 0 | 0 | 0 | 0 |
| 2007-20 | 0 | 0 | 0 | 0 | 0 | 0 | 0 | 0 | 0 | 0 | 0 | 0 |
| 2007-22 | 150 | 152 | 3 | 1 | 0 | 0 | 0 | 0 | 0 | 0 | 0 | 0 |
| 2007-24 | 100 | 170 | 1 | 2 | 20 | 7 | 1 | 1 | 0 | 0 | 0 | 0 |
| 2007-4 | 0 | 0 | 0 | 0 | 150 | 21 | 1 | 1 | 0 | 0 | 0 | 0 |
| 2008-10 | 0 | 0 | 0 | 0 | 30 | 95 | 1 | 1 | 0 | 0 | 0 | 0 |
| 2008-12 | 60 | 11 | 1 | 1 | 0 | 0 | 0 | 0 | 3 | 93 | 1 | 1 |
| 2008-15 | 8 | 322 | 2 | 1 | 0 | 0 | 0 | 0 | 8 | 142 | 1 | 1 |
| 2008-16 | 100 | 12 | 1 | 1 | 0 | 0 | 0 | 0 | 0 | 0 | 0 | 0 |
| 2008-18 | 20 | 88 | 1 | 1 | 0 | 0 | 0 | 0 | 0 | 0 | 0 | 0 |
| 2008-20 | 0 | 0 | 0 | 0 | 100 | 127 | 2 | 2 | 0 | 0 | 0 | 0 |
| 2008-4 | 0 | 0 | 0 | 0 | 100 | 0/257* | 0/3* | 0/1* | 0 | 0 | 0 | 0 |
| 2008-5 | 2 | 16 | 1 | 1 | 0 | 0 | 0 | 0 | 0 | 0 | 0 | 0 |
| 2009-14 | 100 | 1175 | 1 | 1 | 100 | 155 | 1 | 1 | 0 | 0 | 0 | 0 |
| 2009-17 | 150 | 348 | 2 | 1 | 0 | 0 | 0 | 0 | 50 | 162 | 1 | 1 |
| 2009-18 | 300 | 45 | 1 | 1 | 0 | 0 | 0 | 0 | 0 | 0 | 0 | 0 |
| 2009-19 | 120 | 141 | 2 | 1 | 0 | 0 | 0 | 0 | 0 | 0 | 0 | 0 |
| 2009-20 | 300 | 174 | 2 | 2 | 0 | 0 | 0 | 0 | 0 | 0 | 0 | 0 |
| 2009-21 | 50 | 77 | 1 | 1 | 50 | 110 | 1 | 1 | 0 | 0 | 0 | 0 |
| 2010-12 | 0 | 0 | 0 | 0 | 0 | 0 | 0 | 0 | 0 | 0 | 0 | 0 |
| 2011-11 | 300 | 149 | 1 | 1 | 0 | 0 | 0 | 0 | 0 | 0 | 0 | 0 |
| 2011-14 | 175 | 58 | 1 | 1 | 3 | 227 | 1 | 1 | 300 | 60 | 1 | 1 |
| 2013-17 | 0 | 0 | 0 | 0 | 0 | 0 | 0 | 0 | 0 | 0 | 0 | 0 |
| 2013-21 | 100 | 148 | 2 | 1 | 0 | 0 | 0 | 0 | 30 | 169 | 1 | 1 |
| 2016-7 | 300 | 116 | 3 | 2 | 0 | 0 | 0 | 0 | 10 | 0/181* | 0/2* | 0/1* |
| 2017-2 | 300 | 266 | 1 | 1 | 0 | 0 | 0 | 0 | 0 | 0 | 0 | 0 |
| 2017-5 | 0 | 0 | 0 | 0 | 0 | 0 | 0 | 0 | 0 | 0 | 0 | 0 |
| 2019-11 | 13 | 1244 | 1 | 1 | 50 | 3 | 1 | 1 | 4 | 104 | 2 | 2 |
| 2019-7 | 50 | 19 | 1 | 1 | 100 | 5 | 1 | 1 | 7 | 152 | 4 | 2 |
| 2021-12 | 0 | 0 | 0 | 0 | 50 | 160 | 1 | 1 | 150 | 212 | 6 | 3 |
| 2021-15 | 400 | 231 | 1 | 1 | 100 | 5 | 1 | 1 | 100 | 295 | 2 | 1 |
| 2021-4 | 0 | 0 | 0 | 0 | 6 | 252 | 1 | 1 | 0 | 0 | 0 | 0 |
| 2022-11 | 60 | 7 | 1 | 1 | 10 | 32 | 1 | 1 | 0 | 0 | 0 | 0 |
| 2022-3 | 0 | 0 | 0 | 0 | 0 | 0 | 0 | 0 | 0 | 0 | 0 | 0 |
| 2022-4 | 200 | 1448 | 1 | 1 | 0 | 0 | 0 | 0 | 0 | 0 | 0 | 0 |
| 2022-6 | 0 | 0 | 0 | 0 | 2 | 35 | 2 | 2 | 0 | 0 | 0 | 0 |
| 2025-12 | 200 | 209 | 1 | 1 | 200 | 313 | 1 | 1 | 0 | 0 | 0 | 0 |
| 2025-13 | 0 | 0 | 0 | 0 | 20 | 115 | 1 | 1 | 0 | 0 | 0 | 0 |
| 2025-2 | 300 | 132 | 2 | 1 | 0 | 0 | 0 | 0 | 175 | 236 | 2 | 1 |
| 2025-7 | 400 | 164 | 1 | 1 | 300 | 49 | 2 | 1 | 200 | 14 | 2 | 2 |
| 2025-9 | 100 | 11 | 1 | 1 | 10 | 176 | 1 | 1 | 75 | 36 | 1 | 1 |
| 2026-12 | 0 | 0 | 0 | 0 | 100 | 5 | 1 | 1 | 0 | 0 | 0 | 0 |
| 2027-10 | 400 | 0/5* | 0/1* | 0/1* | 10 | 19 | 1 | 1 | 0 | 0 | 0 | 0 |
| 2027-3 | 300 | 171 | 1 | 1 | 0 | 0 | 0 | 0 | 0 | 0 | 0 | 0 |
| 2027-4 | 500 | 244 | 2 | 1 | 0 | 0 | 0 | 0 | 75 | 48 | 1 | 1 |
| 2028-13 | 300 | 194 | 2 | 1 | 0 | 0 | 0 | 0 | 75 | 86 | 1 | 1 |
| 2031-2 | 0 | 0 | 0 | 0 | 0 | 0 | 0 | 0 | 0 | 0 | 0 | 0 |
| 2031-6 | 0 | 0 | 0 | 0 | 0 | 0 | 0 | 0 | 0 | 0 | 0 | 0 |

Samples which have been sequenced twice because of assembly failure with Canu [22] are noticed with an asterisk (*).

Supplementary table 4: number of clones, reads, contigs and unique genes identified per sample and other antibiotic non-susceptibility

|  |  | Antimicrobial non-susceptibility | | | | | | | | | | | | |
| --- | --- | --- | --- | --- | --- | --- | --- | --- | --- | --- | --- | --- | --- | --- |
| Antibiotic | **Chloramphenicol** | | | | | **Trimethoprim-Sulfamethoxazole** | | | | | **Tetracycline** | | | |
| Sample ID | **clones** | **reads** | **contig** | **unique gene sequences** | | **clones** | **reads** | **contig** | **unique gene sequences** | | **clones** | **reads** | **contig** | **unique gene sequences** |
| 2001-11 | 0 | 0 | 0 | | 0 | 100 | 66 | 1 | | 1 | 0 | 0 | 0 | 0 |
| 2001-12 | 0 | 0 | 0 | | 0 | 200 | 0 | 1 | | 1 | 0 | 0 | 0 | 0 |
| 2001-14 | 300 | 215 | 1 | | 1 | 200 | 207 | 1 | | 2 | 300 | 8 | 2 | 1 |
| 2001-7 | 150 | 23 | 1 | | 1 | 0 | 0 | 0 | | 0 | 0 | 0 | 0 | 0 |
| 2001-8 | 20 | 129 | 1 | | 1 | 300 | 148 | 2 | | 2 | 0 | 0 | 0 | 0 |
| 2002-10 | 200 | 62 | 1 | | 1 | 200 | 113 | 1 | | 1 | 0 | 0 | 0 | 0 |
| 2002-5 | 0 | 0 | 0 | | 0 | 100 | 62 | 1 | | 2 | 0 | 0 | 0 | 0 |
| 2003-7 | 0 | 0 | 0 | | 0 | 150 | 118 | 1 | | 2 | 0 | 0 | 0 | 0 |
| 2003-8 | 0 | 0 | 0 | | 0 | 50 | 184 | 1 | | 1 | 0 | 0 | 0 | 0 |
| 2004-13 | 0 | 0 | 0 | | 0 | 150 | 6 | 1 | | 2 | 0 | 0 | 0 | 0 |
| 2005-11 | 0 | 0 | 0 | | 0 | 30 | 161 | 4 | | 2 | 0 | 0 | 0 | 0 |
| 2005-4 | 0 | 0 | 0 | | 0 | 200 | 1171 | 2 | | 2 | 0 | 0 | 0 | 0 |
| 2005-5 | 0 | 0 | 0 | | 0 | 150 | 164 | 1 | | 2 | 0 | 0 | 0 | 0 |
| 2005-6 | 0 | 0 | 0 | | 0 | 200 | 1368 | 1 | | 1 | 0 | 0 | 0 | 0 |
| 2005-8 | 150 | 1698 | 1 | | 1 | 400 | 147 | 2 | | 2 | 0 | 0 | 0 | 0 |
| 2005-9 | 0 | 0 | 0 | | 0 | 0 | 0 | 0 | | 0 | 0 | 0 | 0 | 0 |
| 2006-15 | 0 | 0 | 0 | | 0 | 0 | 0 | 0 | | 0 | 0 | 0 | 0 | 0 |
| 2006-18 | 0 | 0 | 0 | | 0 | 0 | 0 | 0 | | 0 | 0 | 0 | 0 | 0 |
| 2006-2 | 0 | 0 | 0 | | 0 | 200 | 248 | 1 | | 1 | 300 | 21 | 1 | 1 |
| 2006-3 | 0 | 0 | 0 | | 0 | 75 | 78 | 1 | | 1 | 400 | 141 | 1 | 1 |
| 2007-19 | 0 | 0 | 0 | | 0 | 0 | 0 | 0 | | 0 | 5 | 2 | 1 | 1 |
| 2007-20 | 0 | 0 | 0 | | 0 | 0 | 0 | 0 | | 0 | 2 | 135 | 1 | 1 |
| 2007-22 | 100 | 39 | 1 | | 1 | 0 | 0 | 0 | | 0 | 0 | 0 | 0 | 0 |
| 2007-24 | 0 | 0 | 0 | | 0 | 100 | 155 | 1 | | 1 | 0 | 0 | 0 | 0 |
| 2007-4 | 0 | 0 | 0 | | 0 | 50 | 46 | 2 | | 2 | 0 | 0 | 0 | 0 |
| 2008-10 | 0 | 0 | 0 | | 0 | 0 | 0 | 0 | | 0 | 0 | 0 | 0 | 0 |
| 2008-12 | 0 | 0 | 0 | | 0 | 0 | 0 | 0 | | 0 | 0 | 0 | 0 | 0 |
| 2008-15 | 0 | 0 | 0 | | 0 | 0 | 0 | 0 | | 0 | 0 | 0 | 0 | 0 |
| 2008-16 | 0 | 0 | 0 | | 0 | 0 | 0 | 0 | | 0 | 0 | 0 | 0 | 0 |
| 2008-18 | 0 | 0 | 0 | | 0 | 0 | 0 | 0 | | 0 | 0 | 0 | 0 | 0 |
| 2008-20 | 100 | 687 | 2 | | 1 | 0 | 0 | 0 | | 0 | 0 | 0 | 0 | 0 |
| 2008-4 | 0 | 0 | 0 | | 0 | 300 | 24 | 3 | | 1 | 50 | 317 | 1 | 1 |
| 2008-5 | 0 | 0 | 0 | | 0 | 0 | 0 | 0 | | 0 | 0 | 0 | 0 | 0 |
| 2009-14 | 0 | 0 | 0 | | 0 | 0 | 0 | 0 | | 0 | 0 | 0 | 0 | 0 |
| 2009-17 | 0 | 0 | 0 | | 0 | 250 | 836 | 1 | | 2 | 0 | 0 | 0 | 0 |
| 2009-18 | 0 | 0 | 0 | | 0 | 175 | 136 | 2 | | 2 | 0 | 0 | 0 | 0 |
| 2009-19 | 0 | 0 | 0 | | 0 | 150 | 1645 | 2 | | 2 | 0 | 0 | 0 | 0 |
| 2009-20 | 0 | 0 | 0 | | 0 | 300 | 20 | 1 | | 2 | 0 | 0 | 0 | 0 |
| 2009-21 | 0 | 0 | 0 | | 0 | 400 | 646 | 1 | | 2 | 0 | 0 | 0 | 0 |
| 2010-12 | 0 | 0 | 0 | | 0 | 100 | 135 | 1 | | 2 | 0 | 0 | 0 | 0 |
| 2011-11 | 0 | 0 | 0 | | 0 | 300 | 239 | 2 | | 2 | 0 | 0 | 0 | 0 |
| 2011-14 | 0 | 0 | 0 | | 0 | 150 | 188 | 2 | | 2 | 0 | 0 | 0 | 0 |
| 2013-17 | 0 | 0 | 0 | | 0 | 100 | 9 | 1 | | 1 | 0 | 0 | 0 | 0 |
| 2013-21 | 0 | 0 | 0 | | 0 | 0 | 0 | 0 | | 0 | 0 | 0 | 0 | 0 |
| 2016-7 | 0 | 0 | 0 | | 0 | 0 | 0 | 0 | | 0 | 0 | 0 | 0 | 0 |
| 2017-2 | 100 | 37 | 1 | | 1 | 300 | 22 | 1 | | 1 | 0 | 0 | 0 | 0 |
| 2017-5 | 0 | 0 | 0 | | 0 | 150 | 495 | 1 | | 2 | 0 | 0 | 0 | 0 |
| 2019-11 | 30 | 41 | 1 | | 1 | 300 | 176 | 2 | | 2 | 0 | 0 | 0 | 0 |
| 2019-7 | 70 | 76 | 1 | | 1 | 170 | 616 | 1 | | 2 | 0 | 0 | 0 | 0 |
| 2021-12 | 50 | 128 | 1 | | 1 | 200 | 203 | 2 | | 2 | 0 | 0 | 0 | 0 |
| 2021-15 | 100 | 218 | 1 | | 1 | 300 | 233 | 1 | | 2 | 50 | 23 | 1 | 1 |
| 2021-4 | 0 | 0 | 0 | | 0 | 150 | 209 | 1 | | 2 | 0 | 0 | 0 | 0 |
| 2022-11 | 0 | 0 | 0 | | 0 | 300 | 329 | 1 | | 1 | 0 | 0 | 0 | 0 |
| 2022-3 | 0 | 0 | 0 | | 0 | 100 | 2939 | 2 | | 2 | 0 | 0 | 0 | 0 |
| 2022-4 | 0 | 0 | 0 | | 0 | 0 | 0 | 0 | | 0 | 0 | 0 | 0 | 0 |
| 2022-6 | 0 | 0 | 0 | | 0 | 300 | 241 | 1 | | 1 | 0 | 0 | 0 | 0 |
| 2025-12 | 0 | 0 | 0 | | 0 | 0 | 0 | 0 | | 0 | 0 | 0 | 0 | 0 |
| 2025-13 | 0 | 0 | 0 | | 0 | 0 | 0 | 0 | | 0 | 0 | 0 | 0 | 0 |
| 2025-2 | 0 | 0 | 0 | | 0 | 0 | 0 | 0 | | 0 | 0 | 0 | 0 | 0 |
| 2025-7 | 0 | 0 | 0 | | 0 | 0 | 0 | 0 | | 0 | 0 | 0 | 0 | 0 |
| 2025-9 | 0 | 0 | 0 | | 0 | 200 | 115 | 2 | | 2 | 200 | 0/5* | 0/1* | 0/1* |
| 2026-12 | 0 | 0 | 0 | | 0 | 0 | 0 | 0 | | 0 | 0 | 0 | 0 | 0 |
| 2027-10 | 0 | 0 | 0 | | 0 | 400 | 224 | 1 | | 2 | 0 | 0 | 0 | 0 |
| 2027-3 | 0 | 0 | 0 | | 0 | 0 | 0 | 0 | | 0 | 0 | 0 | 0 | 0 |
| 2027-4 | 0 | 0 | 0 | | 0 | 0 | 0 | 0 | | 0 | 0 | 0 | 0 | 0 |
| 2028-13 | 0 | 0 | 0 | | 0 | 0 | 0 | 0 | | 0 | 0 | 0 | 0 | 0 |
| 2031-2 | 50 | 43 | 1 | | 1 | 400 | 578 | 1 | | 2 | 26 | 612 | 1 | 1 |
| 2031-6 | 0 | 0 | 0 | | 0 | 0 | 0 | 0 | | 0 | 50 | 275 | 1 | 1 |

Samples which have been sequenced twice because of assembly failure with Canu [22] are noticed with an asterisk (*).

Figure S1: Number of reads per barcode in the sequencing run No. 1. A) Number of reads after barcode demultiplexing of 2D reads. B) Number of reads used by Canu [22] to build contigs.


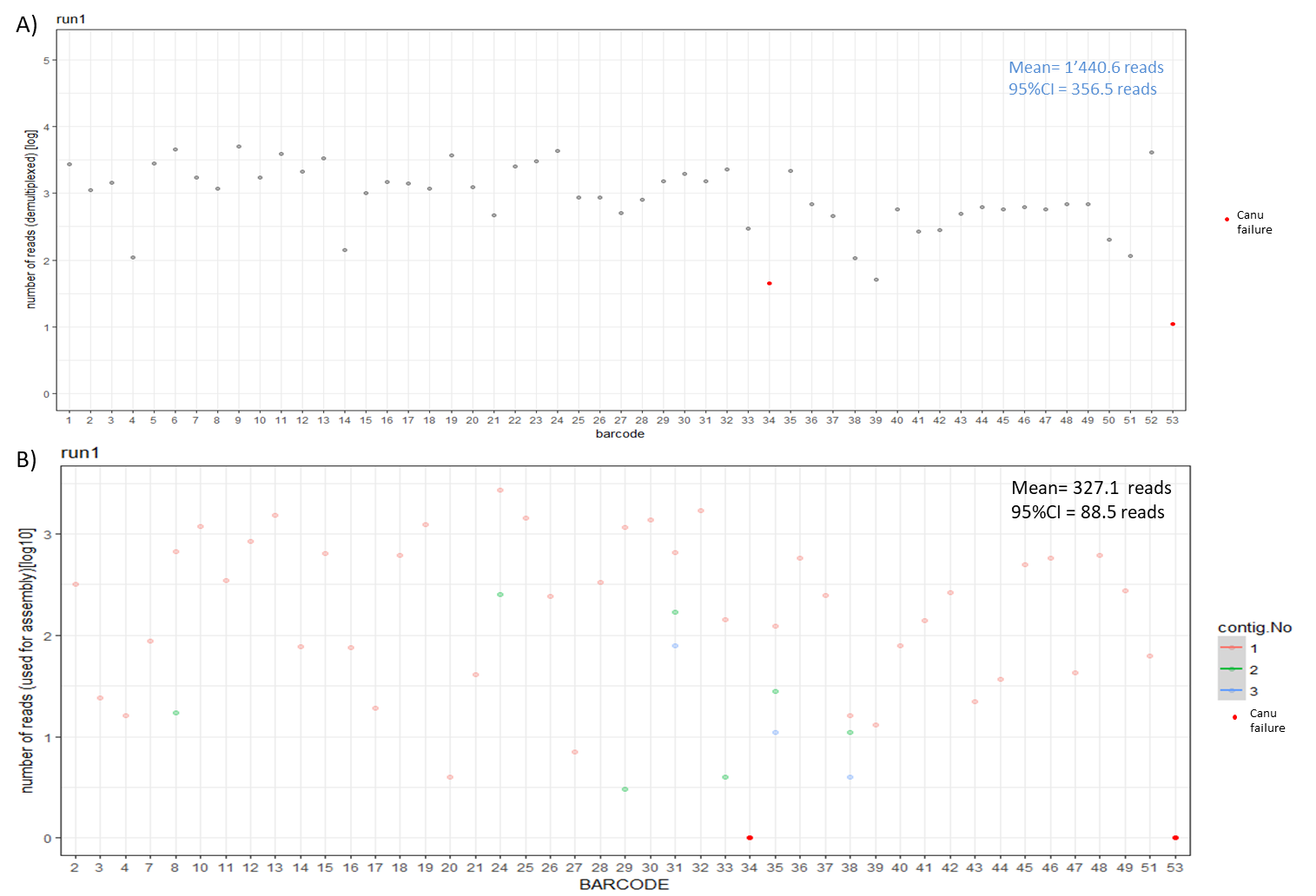


Figure S2: Number of reads per barcode in the sequencing run No. 2. A) Number of reads after barcode demultiplexing of 1D reads. B) Number of reads used by Canu [22] to build contigs.


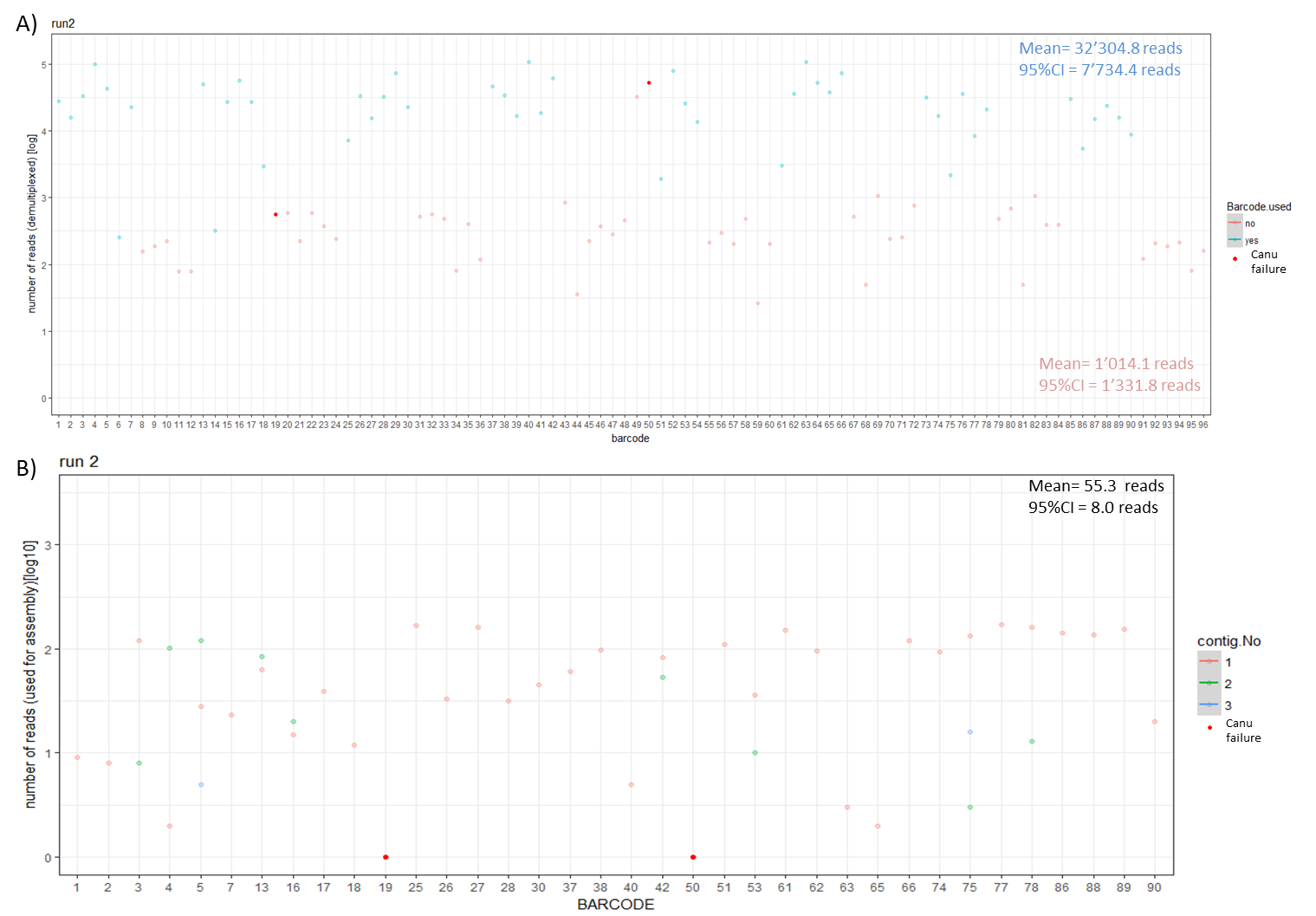


Figure S3: Number of reads per barcode in the sequencing run No. 3. A) Number of reads after barcode demultiplexing of 1D reads. B) Number of reads used by Canu [22] to build contigs.


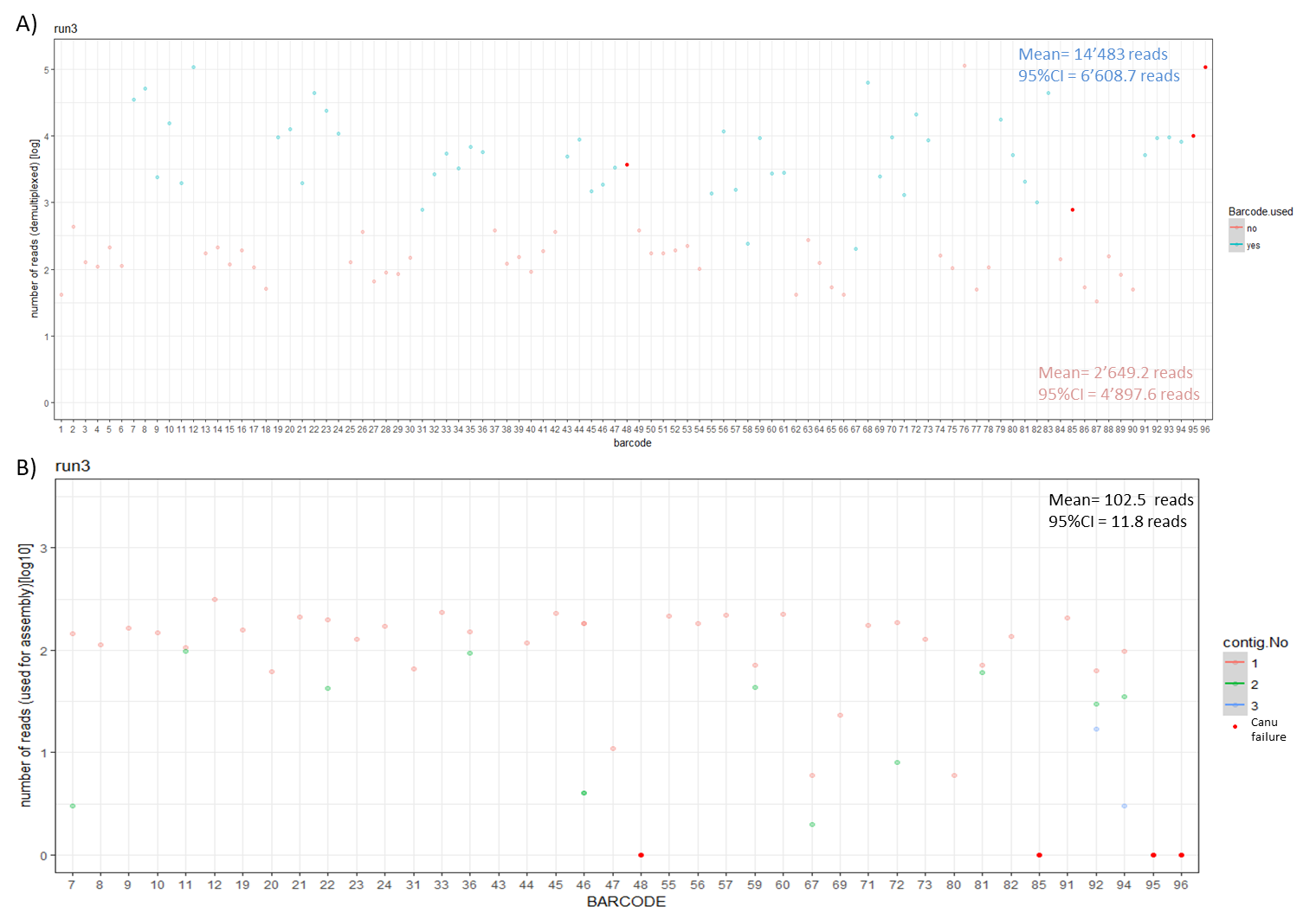


Figure S4: Number of reads per barcode in the sequencing run No. 4. A) Number of reads after barcode demultiplexing of 1D reads. B) Number of reads used by Canu [22] to build contigs.


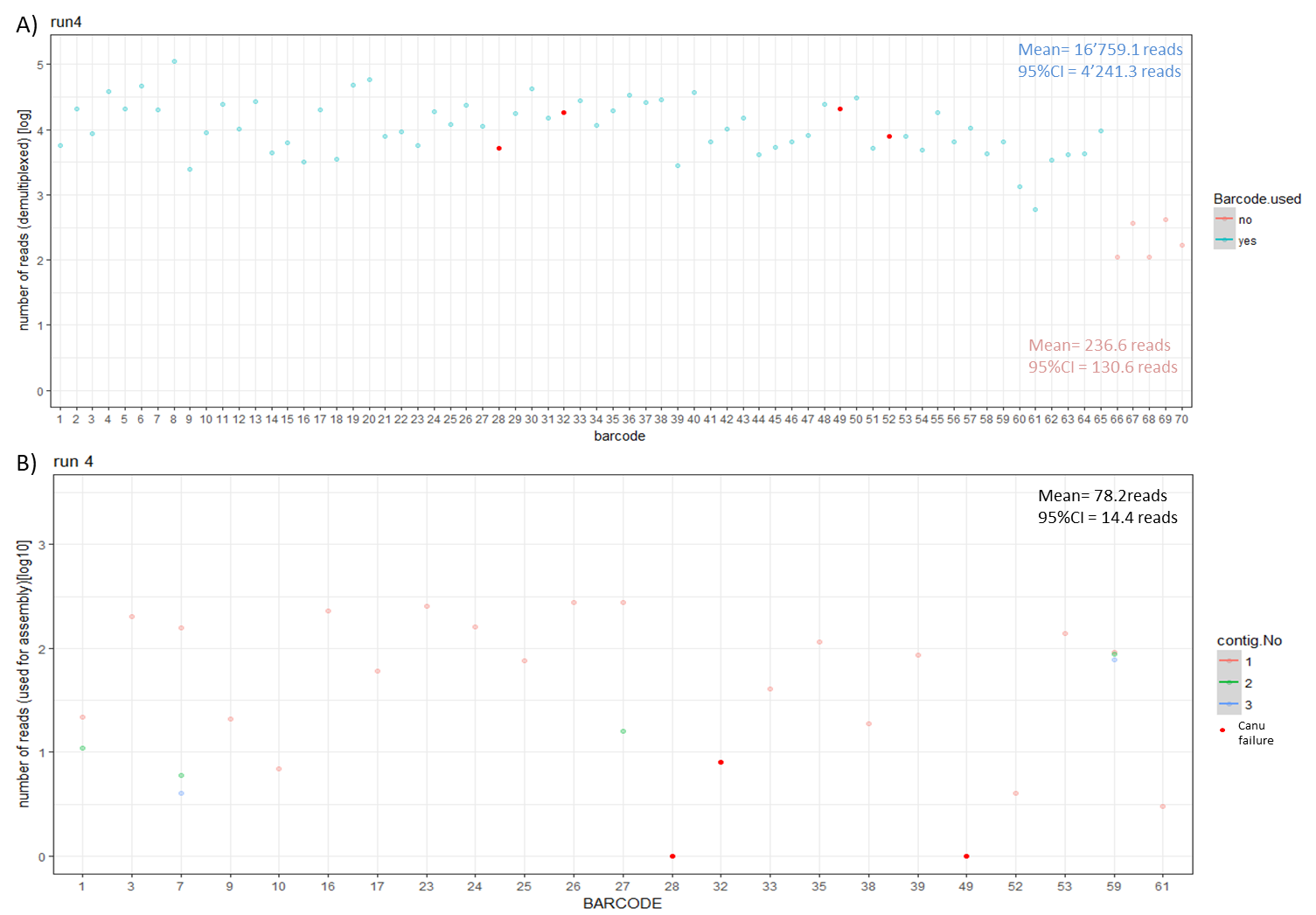


**Figure S5:** Length of contigs. A) Length of all contigs and B) Length of Beta Lactamases are shown in- and excluding Mobile genetic elements (MGE)

**Figure S6:** Effect of mutations rates on ARG identification. To investigate the potential impact of sequencing error rate on the ARG identification, we generated mutant sequences (with different mutation rates) in silico and assessed ARG detection and identification. From a single FASTA sequence (contig obtained after Canu assembly, used for the identification of ARG), we generated hundred FASTA sequences with random point mutations in R (https://github.com/aramette/FastaMutator). Mutations were introduced at the following levels: 1, 2, 3, 5 and 10% of the sequence length. All sequences were then BLAST against the CARD database (online tool, with loose option) and proportions of genes detection were reported. The original sequences coded for: A) a class C β-lactamase (with ESBL phenotype), B) a L1 metallo-β-lactamase, C) blaTEM-type β-lactamase, D) BRO-type β-lactamase, E) OXA-286-like β-lactamase, F) a Cm-acetyltransferase B-type, G) for a Cm efflux system, H) a thymidylate synthase, I) tet(M), J) dfrA3 – a dihydrofolate reductase variant.


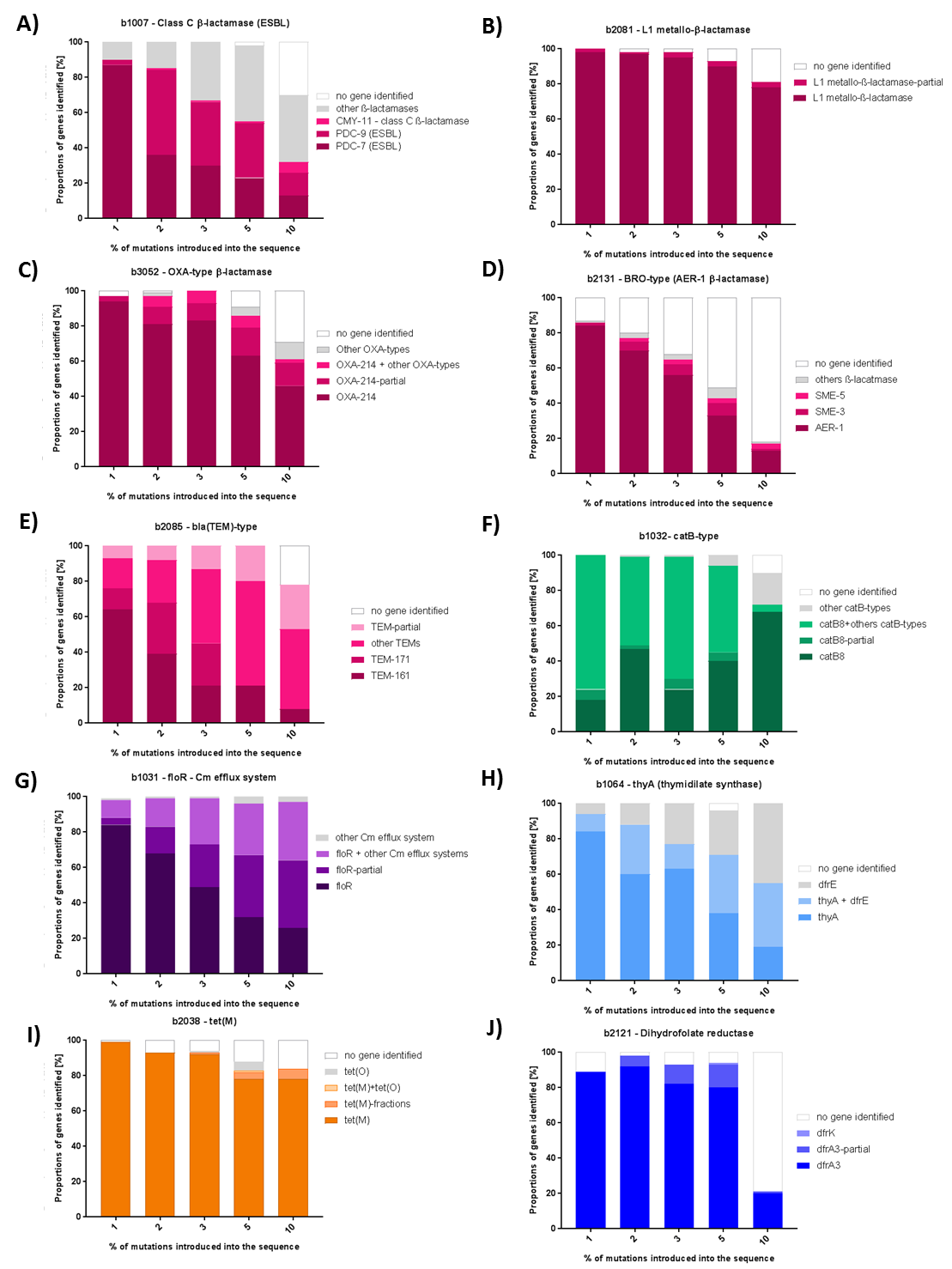

Supplement: Supplementary file 1 [file Table_1.docx]
